# Efficient 2-Step Enzymatic Cascade for the Bioconversion of Oleuropein into Hydroxytyrosol

**DOI:** 10.3390/antiox11020260

**Published:** 2022-01-28

**Authors:** Giorgia Catinella, Silvia Donzella, Gigliola Borgonovo, Sabrina Dallavalle, Martina Letizia Contente, Andrea Pinto

**Affiliations:** Department of Food, Environmental and Nutritional Sciences (DeFENS), University of Milan, Via Celoria 2, 20133 Milan, Italy; giorgia.catinella@unimi.it (G.C.); silvia.donzella@unimi.it (S.D.); gigliola.borgonovo@unimi.it (G.B.); sabrina.dallavalle@unimi.it (S.D.); andrea.pinto@unimi.it (A.P.)

**Keywords:** oleuropein, multi-enzymatic reaction, β-glycosidase, MsAcT, hydroxytyrosol, biocatalysis

## Abstract

Among the plant bioactive components, oleuropein (OLE) is the most abundant phenolic compound in all parts of olive trees (*Olea europaea* L.), particularly concentrated in olive leaves. It has been shown to present various remarkable biological actions, such as antimicrobial, antioxidant, anticancer and anti-inflammatory ones. On the other hand, hydroxytyrosol (HT), the main degradation product of OLE, is considered one of the most powerful antioxidant agents, with higher beneficial properties than the OLE parent compound. In this work, oleuropein was efficiently transformed into hydroxytyrosol using a 2-step biotransformation involving a thermo-halophilic β-glucosidase from *Alicyclobacillus herbarius* (Ahe), which gave the corresponding aglycone with complete conversion (>99%) and rapid reaction times (30 min), and an acyltransferase from *Mycobacterium smegmatis* (MsAcT), here employed for the first time for its hydrolytic activity. After cascade completion, hydroxytyrosol was obtained in excellent yield (>99% m.c., 96% isolated yield) in 24 h. Starting from a natural substrate and employing enzymatic approaches, the final hydroxytyrosol can be claimed and commercialized as natural too, thus increasing its market value.

## 1. Introduction

**Figure 1:**
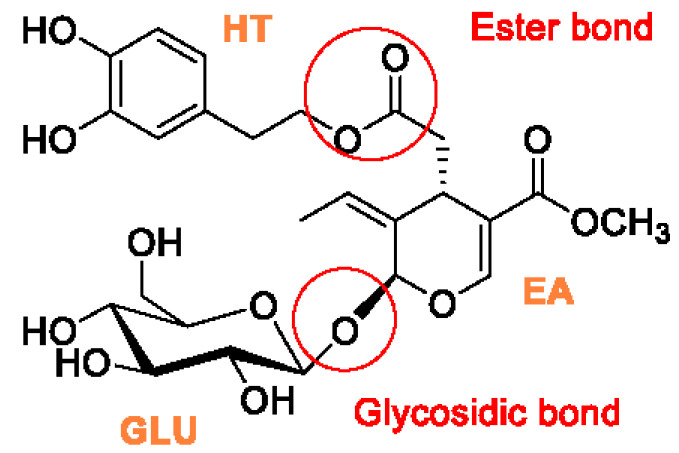
Chemical structure of oleuropein.

Oleuropein (OLE) is the major bioactive antioxidant found in all parts of the olive tree (*Olea europaea* L.), and the most abundant polyphenol in olive leaves, reaching an OLE content of 10–20% of their dry mass [1,2]. It is responsible for the bitter taste of oil and fruits [3]. OLE chemical structure includes an ester bond between elenoic acid (EA) and hydroxytyrosol (HT) and a glycosidic bond between EA and glucose (GLU) [4] (Figure 1). The high interest concerning olive plant phenolic compounds focuses on their involvement in many fields of human health [5,6].

OLE is characterized by various biological actions, among them, the antioxidant, anticancer, antimicrobial and anti-inflammatory activities are noteworthy. It is also known for its hypolipidemic and cardio-protective properties [7,8]. For these reasons it has attracted great interest from the food, cosmetic and pharmaceutical sectors. On the other hand, HT, the main degradation product of OLE, presents superior biological activities than OLE parent compound [5,9,10,11]. Notably, HT is considered the strongest antioxidant agent after gallic acid, and one of the most powerful antioxidants between phenolic compounds from the olive tree, followed by caffeic acid and tyrosol. [12] HT is ten times more active as a free radical scavenger than the well-known green tea, surpassing the antioxidant capacity even of vitamins E and coenzyme Q10 [12,13]. Taking into account that the oxygen radical absorbance capacity (ORAC) assay is considered a common methodology for the determination of antioxidant activity, the ORAC of HT has been evaluated to be 28,000 μmol trolox equivalence (TE)/g [14]. This value is 700-fold higher than that of vitamin C (40 μmol TE/g) [15]. Additionally, HT presents an excellent safety profile: it is a non-mutagenic and non-genotoxic molecule even after the administration of high doses, and no observed adverse effect level (NOAEL) of 500 mg/day (100-times more than the daily recommended dose) has been reported [5,16].

Besides HT, the other OLE degradation products EA and OLE aglycone are gaining increasing attention due to their beneficial properties. In particular OLE aglycone has been demonstrated to exert many activities similar to the ones of oleuropein [17], while EA has been reported as antiviral agent [18].

Even though HT holds promise as a valuable commodity within the pharmaceutical, cosmetic and food industries, its low content in natural sources (less than 0.8% in the olive leaves [7]) triggered the development of various strategies for its preparation.

Many challenges have been encountered in the synthesis of HT, including low final yields, laborious purifications, extensive use of harmful solvents and expensive catalysts [5]. Furthermore, HT is easily oxidized and relatively unstable in the reaction media, thus suitable precautions have to be taken (e.g., absence of strong oxidants, acid pHs). In this contest, biocatalysis has rapidly gained ground in almost every catalytic process due to its advantages, such as selectivity (i.e., regio-, chemo-, stereo-selectivity) higher conversion rates, and low environmental impact when compared to classical synthetic methodology. Such advantages have made the development of biocatalytic approaches a topic of great research interest worldwide.

Until now, a variety of processes for the obtainment of pure HT have been described starting from different cheaper precursors (e.g., dopamine, tyrosol) and employing efficient enzymatic cascades [19,20], while the use of whole cell systems, typically characterized by low substrate loading, has proven difficult for the preparation of HT in high concentrations [13]. Concerning the procedures for the bioconversion of OLE into HT, hydrolytic commercial enzymatic preparations made of lipases, esterases, cellulases, and xylanases have been reported [7]. Although the biocatalytic hydrolysis involved mild conditions, simple operation methods, and final high purity HT, low yields (3–20%) mainly due to the scarce purity of the catalyst, have been observed [7].

The final aim of the study presented here concerns the development of an efficient multi-enzymatic cascade for HT preparation starting from OLE. Enzymatic cascades are considered of high priority when referring to reactions requiring the synergistic action of two or more biocatalysts, such as the bioconversion of oleuropein. Moreover, these systems offer many advantages, among them the possibility of artificially developing metabolic pathways that do not exist in nature, as well as to increase the productivity of well-known biological routes for the preparation of valuable products.

In particular, a 2-step regioselective hydrolysis of OLE has been performed. HT has been obtained in high yields and rapid reaction times employing a halo-thermophilic β-glucosidase from *Alicyclobacillus herbarius* (Ahe) [21], which is able to cleave the glycosidic bond releasing GLU and OLE aglycone, followed by an acyl transferase from *Mycobacterium smegmatis* (MsAcT) [22,23,24,25,26], here exploited for its hydrolytic activity. The superior radical scavenging capacity of HT has been finally evaluated with respect to OLE parent compound and OLE aglycone through DPPH assay.

## 2. Materials and Methods

### 2.1. Materials

Cell growing and strain maintaining media as well as commercially available reagents were purchased from Thermo Fischer Scientific or Merck (Sigma Aldrich, Milan, Italy). Organic solvents and chemical standards were bought from Merck (Sigma Aldrich, Milan, Italy). NMR spectra were recorded on a Brucker Avance 600 MHz spectrometer employing the residual signal of the deuterated solvent as internal standard. Chemical shifts (*δ*) are expressed in ppm and coupling constants (*J*) in Hertz (Hz). Merck Silica gel 60 F254 (aluminum foil) plates were used for TLC analysis (Sigma Aldrich, Milan, Italy); flash column chromatography was performed on Merck Silica gel (230–400 mesh) (Sigma Aldrich, Milan, Italy). Detection of TLC analyses has been performed under UV light at 254 and 365 nm or revealed by a solution of anisaldehyde (2%) and H_2_SO_4_ (1%) in EtOH. HPLC analyses were carried out using a Liquid Chromatograph Varian ProStar, equipped with a ternary pump with a UV-Vis detector Varian Model 345 (both from Varian, Milan, Italy). All the samples were dissolved in ACN:H_2_O 1:1 to stop the reaction and filtered with 0.45 μm nylon filters. The commercial kit “DPPH Antioxidant Capacity Assay” for the analysis of the free radical scavenging capacity was bought from Bioquochem, Asturie, Spain.

### 2.2. Expression and Purification of Ahe

Enzyme preparation including protein expression and purification were performed as previously described by Delgado et al. 2021 [21]. Ahe has been expressed in very good yield (70–100 mg/L of culture), and analyzed by SDS page (See Supplementary Information, Figure S1).

### 2.3. Ahe Activity Assay

Activity measurements have been performed as reported by Benitez-Mateos and Contente, 2021 [27]. The specific activity (U/mg) was expressed as µmol of product generated per minute per milligram of enzyme. Ahe final specific activity: 20 U/mg.

### 2.4. Biotransformation of OLE to the Corresponding Aglycone

In 25 mL screw cap tubes, 5 mL reactions using Ahe were performed. Oleuropein (10 mg/mL) was solubilized in H_2_O:EtOH 80:20. After the addition of Ahe (1 mg/mL), the reaction mixture was left under magnetic stirring at 35 °C and monitored by TLC (EtOAc:Acetone:H_2_O:Acetic acid 7:2.5:0.8:0.2 or DCM:MeOH 98:2) at different reaction times (5 min, 15 min, 30 min). Complete conversion into the corresponding oleuropein aglycone was obtained after 30 min. Extraction with EtOAc (10 mL × 3) was subsequently performed and the organic phase collected, dried over Na_2_SO_4_, filtered and evaporated under reduced pressure. The crude extract was analyzed by 1D and 2D NMR, identifying the characteristic signals of oleuropein aglycone (OA) and its aldehydic forms (AOA and dialdehydic form) by comparison with the literature [28,29] (See Supplementary Information, Figures S6–S8). The crude mixture was checked through HPLC (see below) and used without any further purification for the second step.

### 2.5. Expression and Purification of MsAcT

Enzyme preparation including protein expression and purification were performed following the procedure previously described by Contente et al. 2018 [24]. MsAcT has been expressed in excellent yield (150–200 mg/L of culture), and was analyzed by SDS page (See Supplementary Information, Figure S2).

### 2.6. MsAcT Activity Assay

Activity measurements were performed as reported by Contente et al., 2018 [24]. The specific activity (U/mg) was expressed as µmol of product generated per minute per milligram of enzyme. MsAcT final specific activity: 120 U/mg.

### 2.7. Biotransformation of Oleuropein Aglycone to Hydroxytyrosol

Batch reactions were performed in 10 mL screw cap tubes; 4 mL reaction mixture in 100 mM phosphate buffer, pH 8.0, containing 10 mg/mL oleuropein aglycone and 1 mg/mL enzyme was left under magnetic stirring at 30 °C. Samples withdrawn at different reaction times (30 min, 1 h, 3 h, 6 h, 24 h) were monitored by TLC (DCM/MeOH 9:1 revealed by anisaldehyde). After 24 h, complete conversion was reached; the reaction mixture was quenched with NaHCO_3_ 5% and extracted with EtOAc (5 mL × 3). The organic phases were collected, washed with brine (5 mL), dried over Na_2_SO_4_, filtered and evaporated under reduced pressure. The crude extract was subsequently purified by silica gel column chromatography (2-MeTHF:AcOEt 1:1) finally obtaining 15.5 mg of pure HT (96% isolated yield). Purity of HT was checked by HPLC analysis (see below). 

^1^H NMR (600 MHz, (CD_3_)_2_CO) *δ*: 7.66 (2H, brs), 6.70 (1H; brs), 6.69 (1H, dd, *J* = 8.0, 2.8), 6.53 (1H, dd, *J* = 8.0, 2.8), 3.69 (2H, t, *J* = 7.3), 2.64 (2H, t, *J* = 7.3).

^13^C NMR (150 MHz, (CD_3_)_2_CO) *δ*: 145.8, 144.2, 132.0, 121.1, 116.9, 116.0, 64.3, 39.8.

### 2.8. HPLC Analysis

50 µL aliquots were quenched with ACN in the first step and with NaHCO_3_ 5% in the second one at different reaction times (5 min, 15 min, 30 min, 1 h, 3 h, 6 h, 24 h) to stop the reaction. Samples were properly diluted in the mobile phase employed for the analyses and were filtered using 0.45 μm nylon filters. HPLC runs were performed with RP 18 (Hypersil ODS, Thermo, 5 μm 300 × 4 mm i.d.), fitted to a HPLC Varian ProStar, equipped with a ternary pump with a UV-Vis detector Varian Model 345 (both from Varian, Milan, Italy). Analyses were carried out using the following gradient: 90/10 (*v/v*) H_2_O milliQ/ACN until 60% of ACN for 15 min (t_0min_ → t_15min_) and maintaining 60% ACN for 25 min (t_15min_ → t_40min_). Flow rate: 1 mL/min, λ = 210 nm (See Supplementary Information, Figures S3–S5).

### 2.9. DPPH Radical Scavenging Assay

Measurements of DPPH radical scavenging activity of OLE, the corresponding aglycone and HT were performed using a commercial kit, following the manufacturer’s instructions. Briefly, samples were appropriately diluted in DMSO and mixed with the DPPH solution provided by the kit. The total antioxidant capacity (TAC) was determined by measuring absorbance at 517 nm through a spectrophotometer (Eppendorf, Milan, Italy) and calculating the corresponding percentage of the inhibition of the radical DPPH as reported in the kit instructions.

## 3. Results and Discussion

In the present work, a robust biocatalytic procedure for the production of HT from oleuropein was developed. More specifically, the halo-thermophilic β-glucosidase Ahe and MsAcT acyltransferase, here exploited for its hydrolitic properties, were successfully home-prepared and employed for a green, safe and high-yielding production of natural HT.

### 3.1. Preparation of Ole Aglycone through Ahe β-Glucosidase

β-Glucosidases (EC 3.2.1.21) represent a group of enzymes that are able to hydrolyze terminal non-reducing glycosyl residues from glycosides [30]. They are acquiring high importance due to the wide range of biotechnological applications in food, cosmetic, pharmaceutical and agricultural sectors, among others. They have been successfully applied in industrial processes [31], becoming key tools in the hydrolysis of very stable glycosidic bonds in a selective, clean and efficient way. Although industrial methodologies based on the employment of β-glucosidases are typically less energy-consuming, environmentally-friendly and safer than the chemical ones, they are usually characterized by harsh conditions such as low pH, high sugar concentration, the presence of organic solvents and high temperature, leading to enzymatic deactivation [32,33]. Several strategies can be adopted to enhance the biocatalyst stability such as rational or random evolution [26,34], immobilization techniques [35] as well as the research for novel and more industrially-suitable enzymes. In this context, extremophilic microorganisms, well adapted to leave in drastic conditions, represent an alternative source of enzymes which are more resistant to industrial processes than the mesophilic counterparts [36].

**Figure 2:**
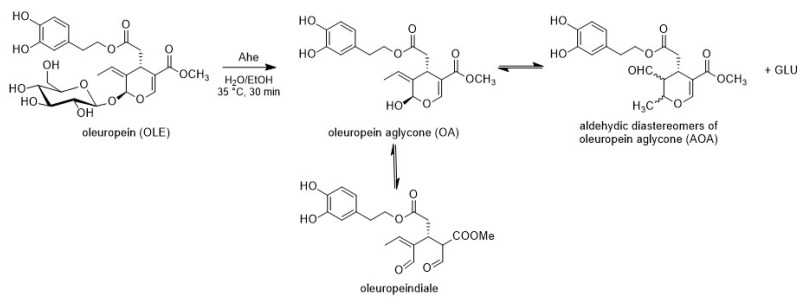
Biotransformation of oleuropein.

After a screening of different thermo-acidophilic β-glucosidases (HOR from *Halothemothrix orenii*, Aac from *Alicyclobacillus acidophilus*) a better performance of the one from *Alicyclobacillus herbarius* (Ahe 1 mg/mL) in the preparation of OLE aglycone from oleuropein as starting material was observed (Figure 2, See Supplementary Information, Table S1).

After 30 min, high concentration of the substrate (10 g/L) has been completely biotransformed in the desired product. Notably, previous attempts to prepare OLE aglycone employing commercially available β-glucosidases required longer reaction times (20–24 h) [1,7]. Among the mesophilic β-glucosidases described so far, many of them are inhibited by both organic solvents and glucose, which would be present at increasing concentrations as the hydrolytic process progresses. The high stability of the extremophilic Ahe to the ethanol employed to maximize the substrate solubility (20% *v/v*) and the high sugar accumulation is noteworthy. After HPLC and NMR analyses, the final product has been used for the second step without any further purification.

### 3.2. Preparation of Hydroxytyrosol

For the following step, an acyl transferase from *Mycobacterium smegmatis* (MsAcT), a well-known biocatalyst produced in our lab [24], has been employed. So far MsAcT has been reported for its capability to efficiently catalyze condensation reactions (i.e., formation of esters and amides) in water and organic solvents both in batch [6,24,37,38] and in continuous mode [20,39,40,41,42,43,44].

MsAcT presents common characteristics of the SGNH superfamily of serine-hydrolases, including the Ser-His-Asp catalytic triad [22] which is evolutionary well conserved. More specifically, in the GDSL-like family which includes hydrolytic enzymes (e.g., peptidases, lipases, esterases) as well as MsAcT, both catalytic serine and histidine are highly conserved [26].

**Figure 3:**
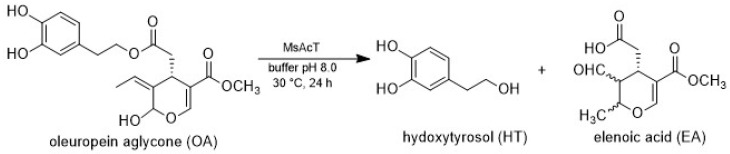
Biotransformation of oleuropein aglycone into hydroxytyrosol.

Taking into consideration the MsAcT mechanism of action [22], by simply avoiding the use of acyl donors and working in water-media, the enzyme has been exploited here for the first time for its hydrolytic activity instead of its condensation capability. OLE aglycone (10 g/L) was efficiently transformed into the corresponding HT (>99% m.c., 96% isolated yield) in 24 h using 1 mg/mL of the catalyst (Figure 3). Notably, no HT formation was observed employing *Candida antarctica* lipase B (i.e., CAL B) one of the most known lipases, while direct cleavage of the ester bond without the first intervention of Ahe (i.e., GLU removal) failed with both MsAcT and CAL B, probably due to the high hydrophobicity of the active sites which are not able to accommodate the sugar moiety. Previously reported attempts to produce HT from OLE aglycone mainly rely on drastic reaction conditions (i.e., high temperature and long reaction times) [1,7], often giving rise to multiple products thus complicating the purification steps. Here, HT was obtained as a single product, allowing for an easier and faster purification procedure.

Although protein purification is often considered a cost and time-consuming technique, here the advantages of using pure enzymes with respect to recombinant whole cells overcome its limitations. Firstly, an intimate contact between the starting materials and the catalysts is guaranteed as no physical barrier to substrates and products represented by the cellular membrane is present; this not only allowed for high substrate loading but also to fast reactions; all unnecessary pathways and energy draining processes typical of a cell deeply impacting on the overall final yield are here avoided, giving rise to more selective and highly productive biotransformations. Furthermore, the reactions here presented do not need cofactors, which is one of the main reasons for using microbial machinery systems. Considering the high stability of both Ahe and MsAcT employed in the 2-step biotransformation (more than one month of storage without any loss of activity) as well as the high purification yields, just two purification rounds were necessary for the optimization of the OLE-to-HT cascade, making this strategy also appealing from an economic point of view.

### 3.3. Determination of the Free Radical Scavenging Capacity

To confirm the radical scavenging capacities of HT and its parent compounds OLE and OLE aglycone, an easy and highly reproducible DPPH spectrophotometric test was performed (Table 1). 

As shown in Table 1, HT was demonstrated to be roughly 2-fold more active as free radical scavenger than OLE starting material and to have higher antioxidant capacity when compared to OLE aglycone. The ability to transfer a hydrogen from its phenolic OH groups to reactive oxygen species, minimizes the damage operated by oxidative processes implicated in the development of many diseases such as inflammation, cardiovascular diseases, diabetes, cancer and ageing [13] as well as oxidative deterioration in foodstuff [45]. While HT anti-cancerogenic and anti-diabetic effects have been already demonstrated [46,47] as well as its food preservative properties [48], in vivo studies have shown that HT supplementation inhibits the efflux of brain cell death markers, making this compound a potential neuroprotectant [49].

## 4. Conclusions

From a circular economy point of view, the collection and re-use of agri-food waste and residues deeply impact both society and the environment. In this contest, olive tree cultivation residues could represent a cheap source of natural bioactives. These molecules, once biocatalytically processed, can generate high valuable compounds which can be commercialized as natural, following the FDA and EMA regulation. In particular, oleuropein, the main component of olive leaves, was efficiently transformed using the highly-stable β-glucosidase from *Alicyclobacillus herbarius* followed by MsAcT, here exploited for the first time for its hydrolytic properties. In the first step, OLE aglycone was obtained with excellent yields (>99% m. c.) and rapid reaction times (30 min), while in the second one, hydroxytyrosol, one of the most potent natural antioxidants, was generated as a single product with complete conversion in 24 h. Previous attempts to employ β-glucosidases with OLE as substrate required longer reaction times (20–24 h), while the production of HT from OLE aglycone mainly rely on drastic thermic treatments, giving rise to multiple by-products thus complicating the purification steps. The HT superior free radical scavenging capacity was finally confirmed through DPPH assay, resulting roughly 2-times more active than OLE starting material.

Having an efficient and robust strategy for the preparation of HT is of high priority due to its superior beneficial properties when compared to OLE parent compound and its lower content in natural sources.

Moreover, whereas HT is considered as GRAS (Generally Recognized as Safe) by the EFSA and FDA, there is no official GRAS status for OLE pure form.

The claimed health-promoting properties of HT, allied to the easy and affordable 2-step enzymatic preparation here proposed, make this compound an excellent ingredient for a variety of applications in the food, pharmaceutical and cosmetic industry. 

## Figures and Tables

**Table 1:** Determination of the free radical scavenging capacity.

Compound	DPPH Radical Inhibition (%) *
OLE	25
OLE aglycone	27
HT	44

* The results are showed as the average of two different measures.

## Data Availability

Data are contained within the article and supplementary material.

## Supplementary Materials

The following are available online at https://www.mdpi.com/article/10.3390/antiox11020260/s1, Figure S1: SDS page Ahe, Figure S2: SDS Page MsAcT, Figures S3–S5: HPLC analysis, Figures S6–S8: 1H NMR of oleuropein aglycone, Figures S9 and S10: 1H and 13C NMR of hydroxytyrosol.
