# Supplementary material for: Efficient 2-Step Enzymatic Cascade for the Bioconversion of Oleuropein into Hydroxytyrosol
Source: Antioxidants (Basel). 2022 Jan 28;11(2):260. doi: 10.3390/antiox11020260 (PMC8868057; doi:10.3390/antiox11020260)
Supplement: Supplementary file 1 [file antioxidants-11-00260-s001.zip › antioxidants-1544954-supplementary.pdf]

Supplementary Information

Efficient 2-Step Enzymatic Cascade for the Bioconversion of Oleuropein into Hydroxytyrosol

Giorgia Catinella[1], Silvia Donzella[1], Gigliola Borgonovo[1], Sabrina Dallavalle[1], Martina Letizia Contente[1*], and Andrea Pinto[1]

[1]Department of Food, Environmental and Nutritional Sciences (DeFENS), University of Milan, Via Celoria 2, 20133 Milan, Italy

*Correspondence: martina.contente@unimi.it; Tel.: +390250316817

Table of contents

SDS page Ahe 3

SDS page MsAcT 4

Comparison between different halo-termophilic β-glucosidases 5

HPLC analysis 6

1D and 2D NMR spectra 7

Bibliography 12

1. **SDS page Ahe**

Ahe has been expressed in very good yield (70-100 mg/L of culture), purified by affinity chromatography and analyzed by SDS page. Ahe has a molecular weight of 52 KDa.

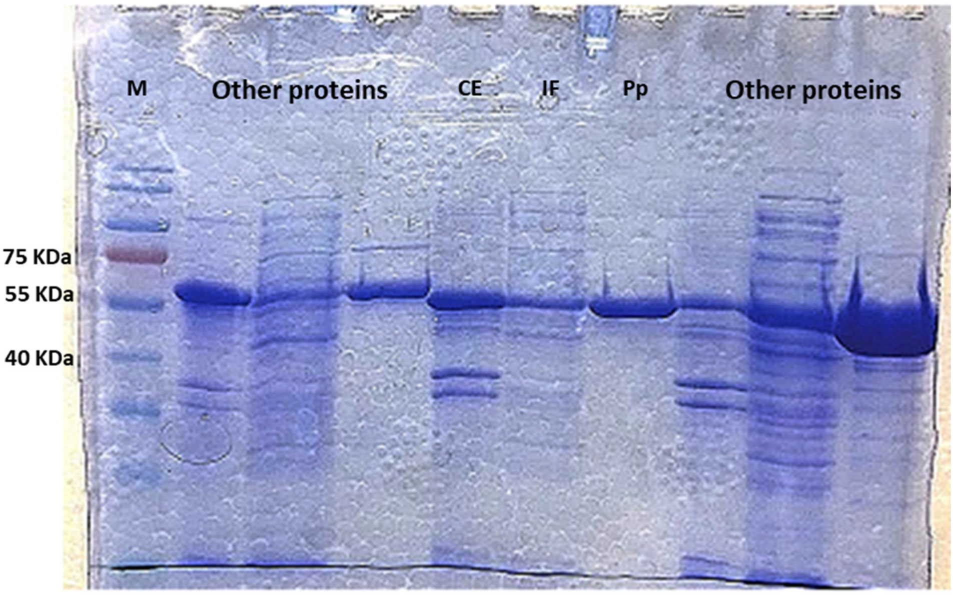

Figure S1. SDS-PAGE with the purification of Ahe. M: molecular weight marker; CE: crude extract after sonication and centrifugation; IF: Insoluble fraction after sonication, Pp: Pure protein.

2. SDS page MsAcT

MsAcT has been expressed in excellent yield (160-190 mg/L of culture), and analyzed by SDS page. MsAcT has a molecular weight of 25.6 KDa.

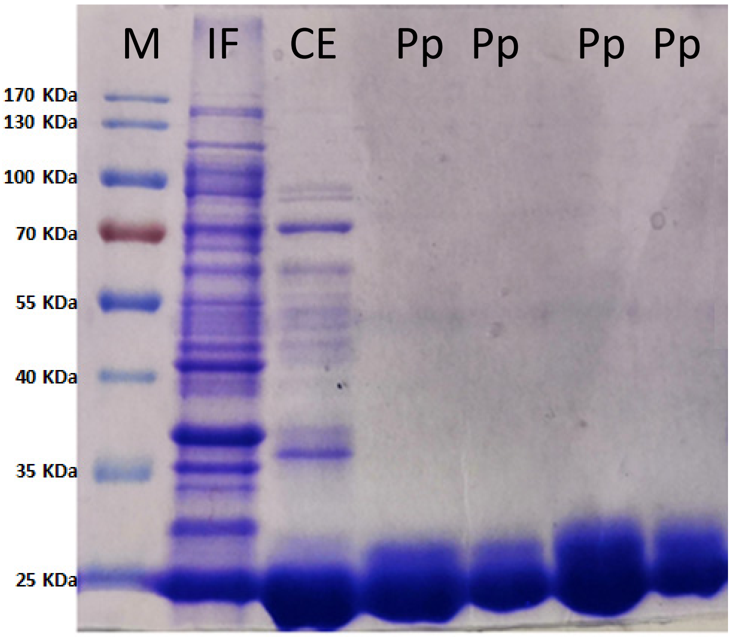

Figure S2. SDS-PAGE with the purification of MsAcT. M: molecular weight marker; IF: Insoluble fraction after sonication, CE: crude extract after sonication and centrifugation; Pp: Pure protein.

3. Comparison between different halo-termophilic β-glucosidases

Table S1. Comparison between three different β-glucosidases in the biotransformation of OLE to the corresponding aglycone.

Biocatalyst	Reaction time	Molar conversion (%)
Ahe (*Alicyclobacillus herbarius*)	30 min	99
HOR (*Halothemothrix orenii*)	30 min	70
	1 h	80
	3 h	98
Aac (*Alicyclobacillus acidophilus*)	30 min	20
	1 h	30
	3 h	50
	6 h	60
	24 h	60

4. HPLC analysis

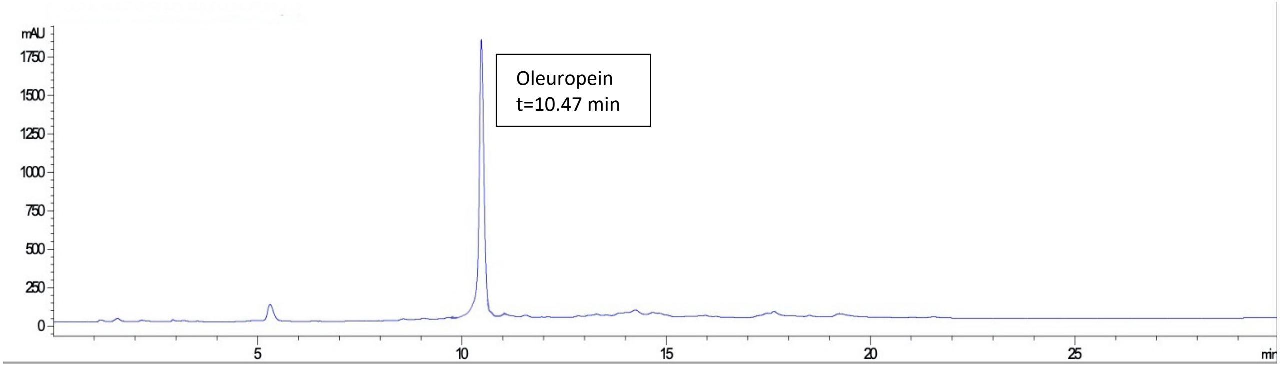

Figure S3. HPLC of oleuropein (OLE) at λ= 210 nm (t=10.47 min).

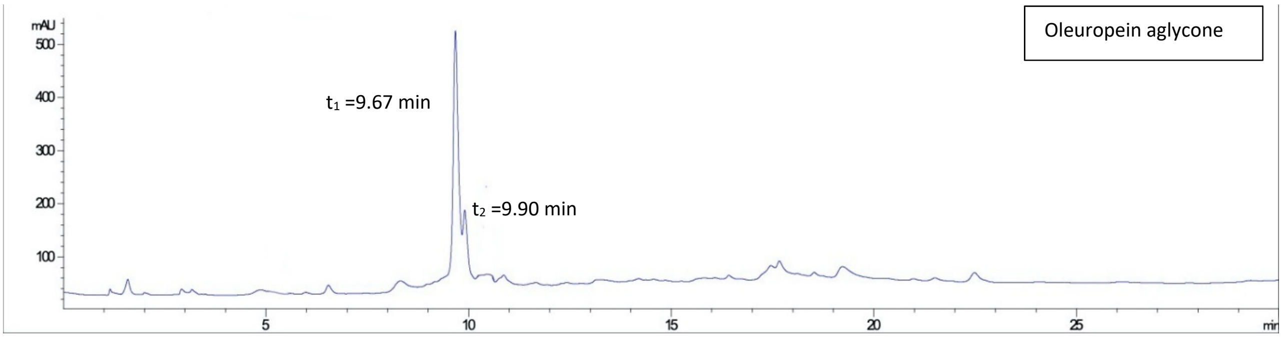

Figure S4. HPLC of biotransformation from OLE to oleuropein aglycone using Ahe after 30 min (λ= 210 nm).

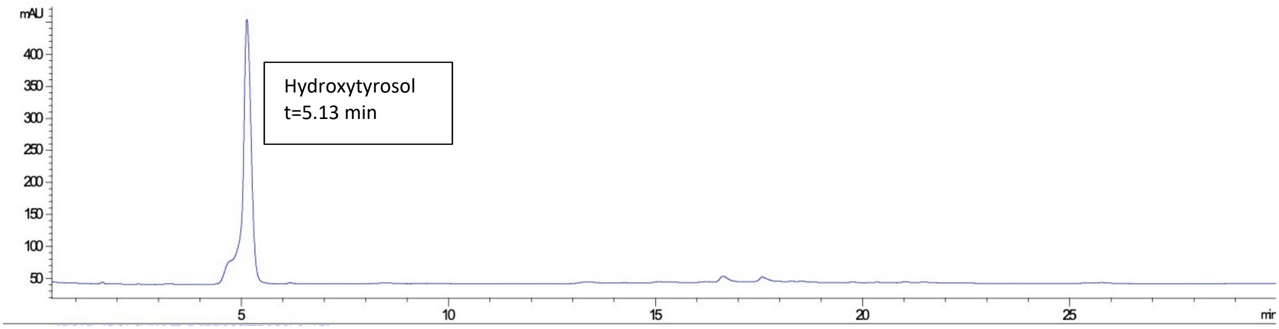

Figure S5. HPLC of hydroxytyrosol (HT) at λ= 210 nm (t=5.13 min).

5. ^{1}H NMR of oleuropein aglycone

In the ^{1}H NMR crude reaction mixture can be identified oleuropein aglycone (OA) and its aldehydic forms (AOA) composed of three major diastereomers, (5S, 8R, 9S), (5S, 8S, 9S) and (5S, 8R, 9R) and the opened aldehydic form (oleuropeindiale).

To confirm the results and to assign each signal to the corresponding aldehydic form, we performed ^{13}C NMR and 2D NMR in CD_3CN. The results are consistent with those reported in the literature [1,2].

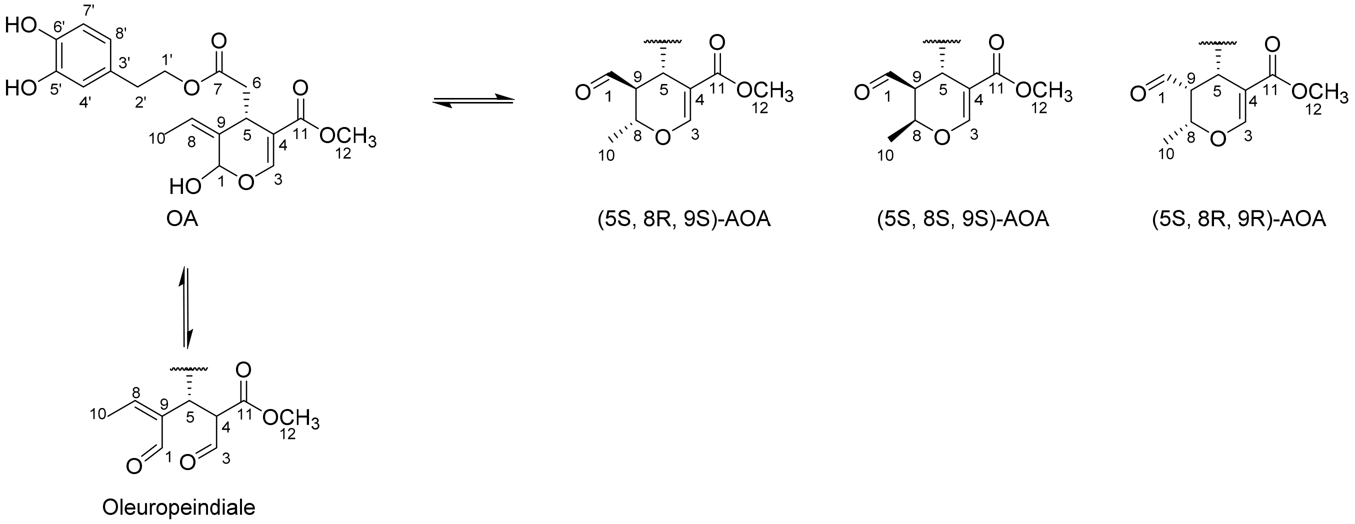

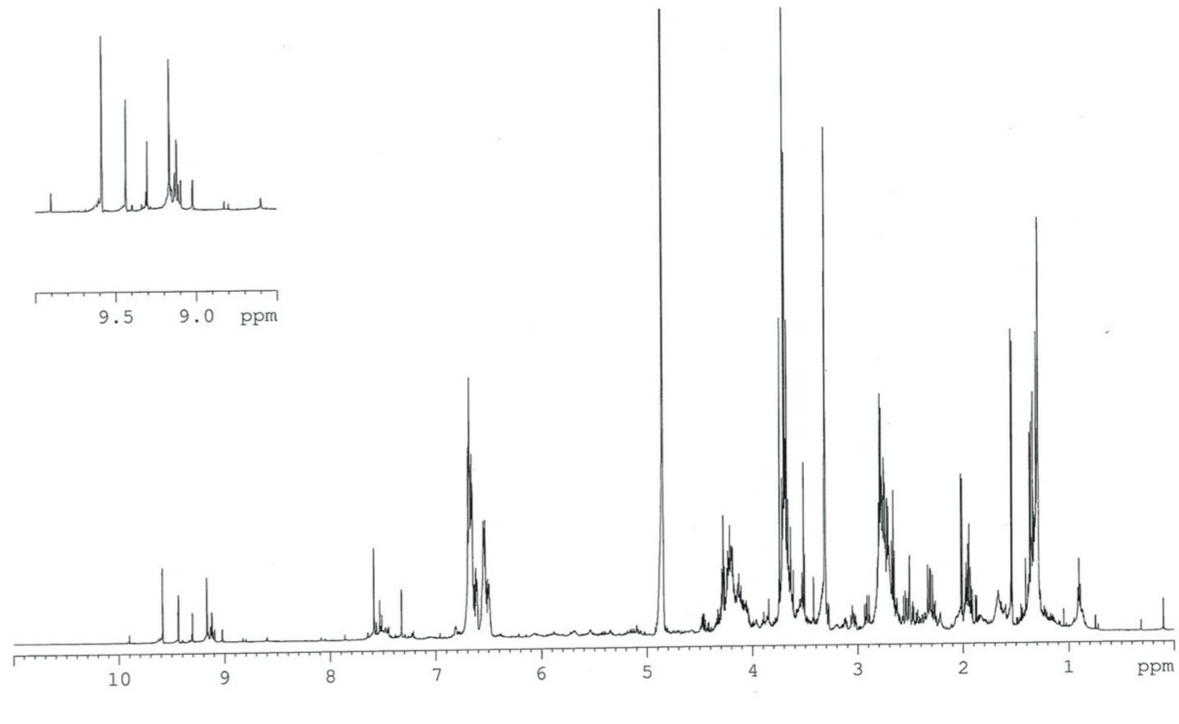

Figure S6. ^{1}H NMR in CD_3OD of oleuropein aglycone.

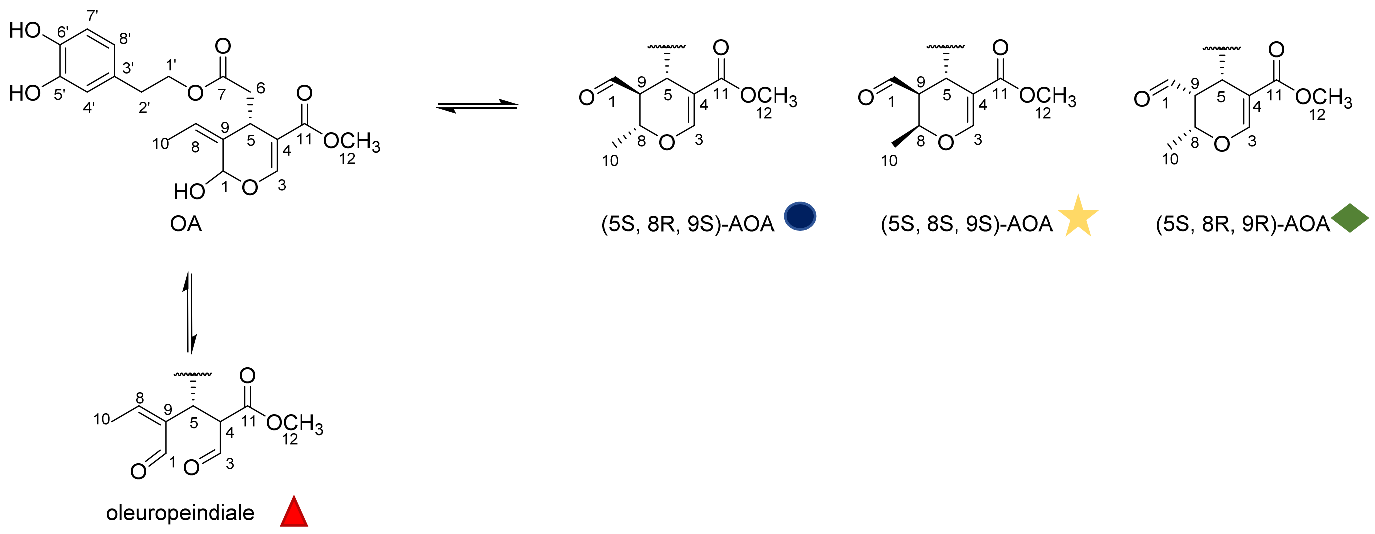

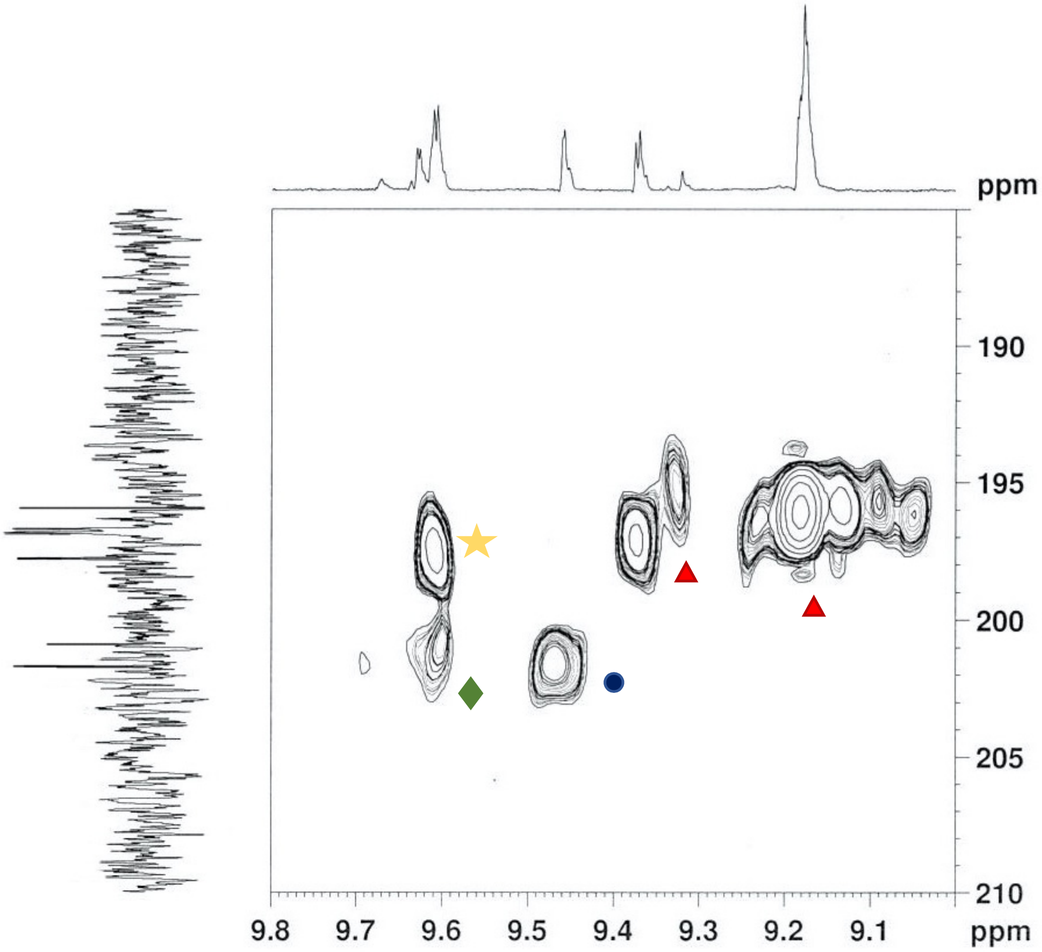

Figure S7. Zoom of HSQC NMR of oleuropein aglycone in CD_3CN.

Name	^{1}H NMR	^{13}C NMR
(5S, 8R, 9S)-AOA	9.46	201.7
(5S, 8S, 9S)-AOA	9.61	197.49
(5S, 8R, 9R)-AOA	9.62	200.9
dialdehydic form (oleuropeindiale)	9.40<br>9.17	197.07<br>195.95

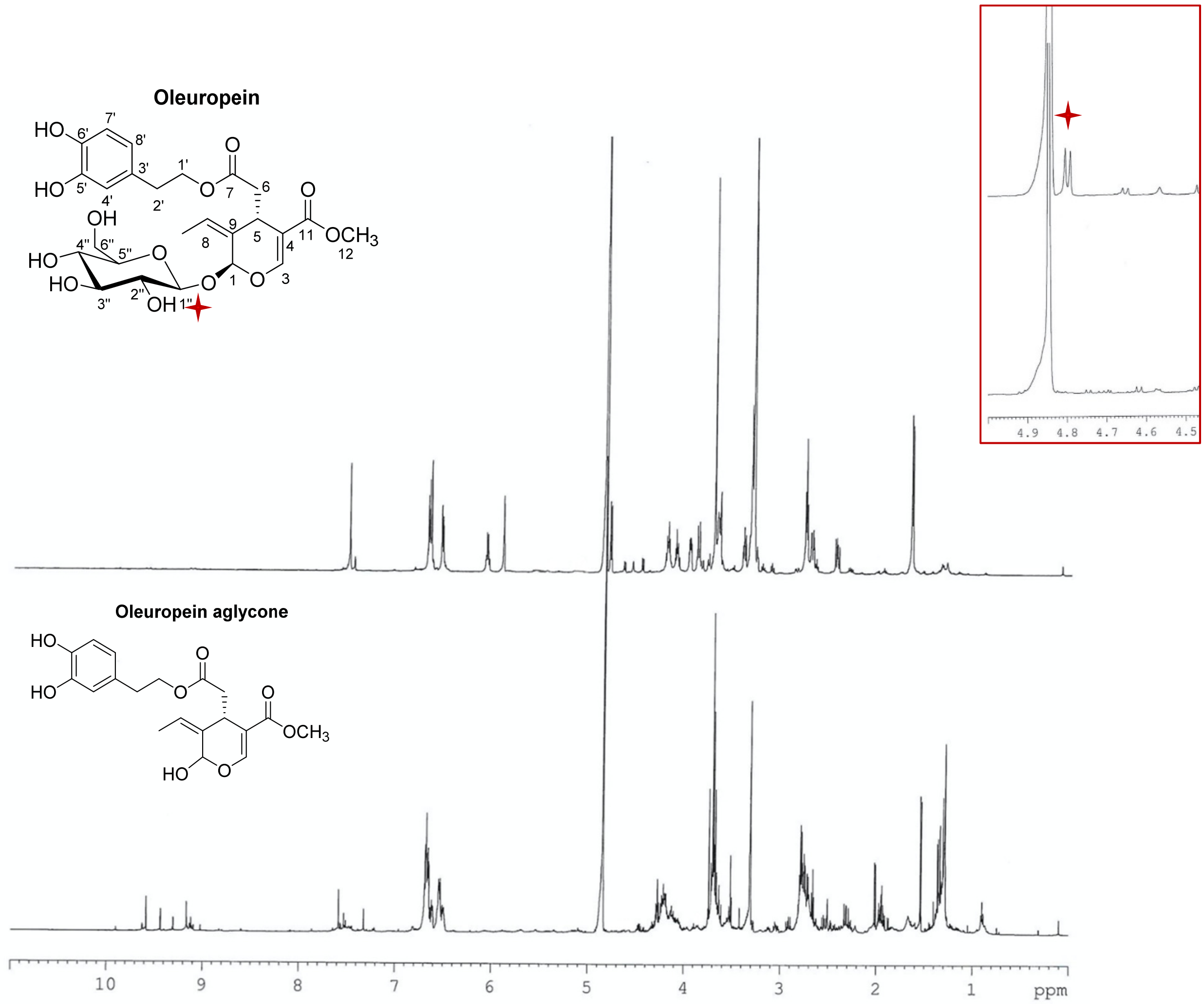

Figure S8. Comparison of ^{1}H NMR in CD_3OD oleuropein and oleuropein aglycone. Zoom of anomeric hydrogen of oleuropein.

6. ^1H and ^{13}C NMR of hydroxytyrosol

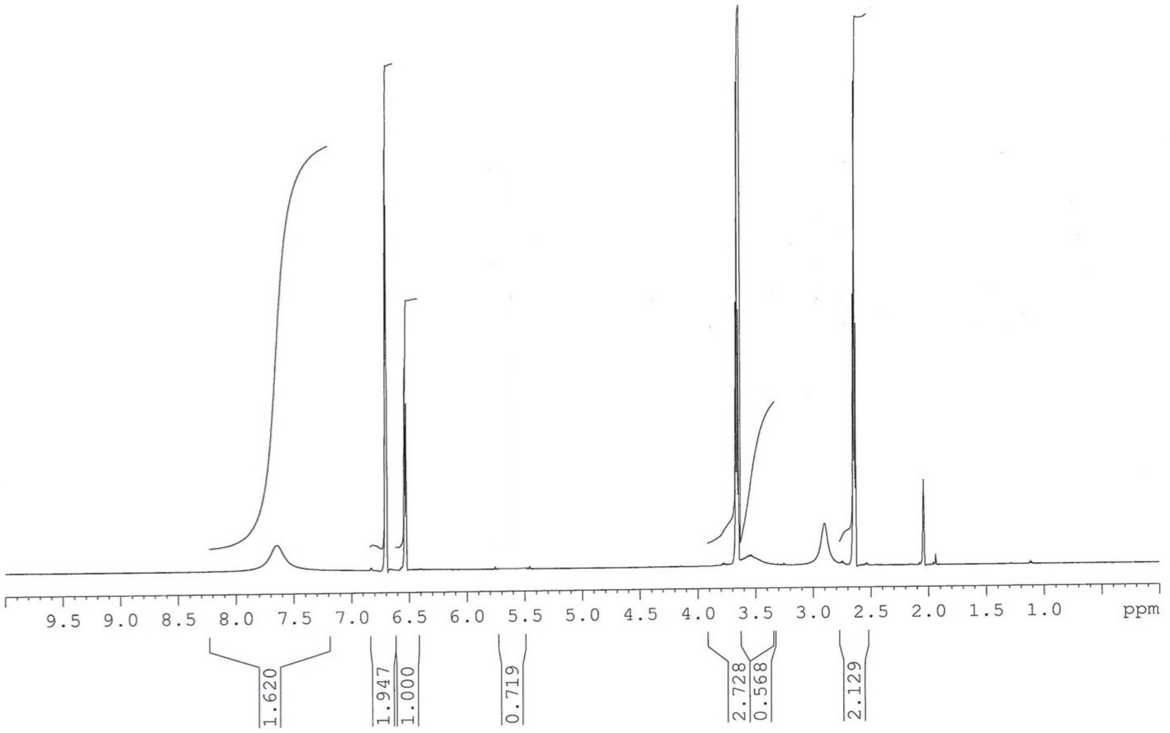

Figure S9. ^1H NMR in $(CD_3)_2CO$ hydroxytyrosol

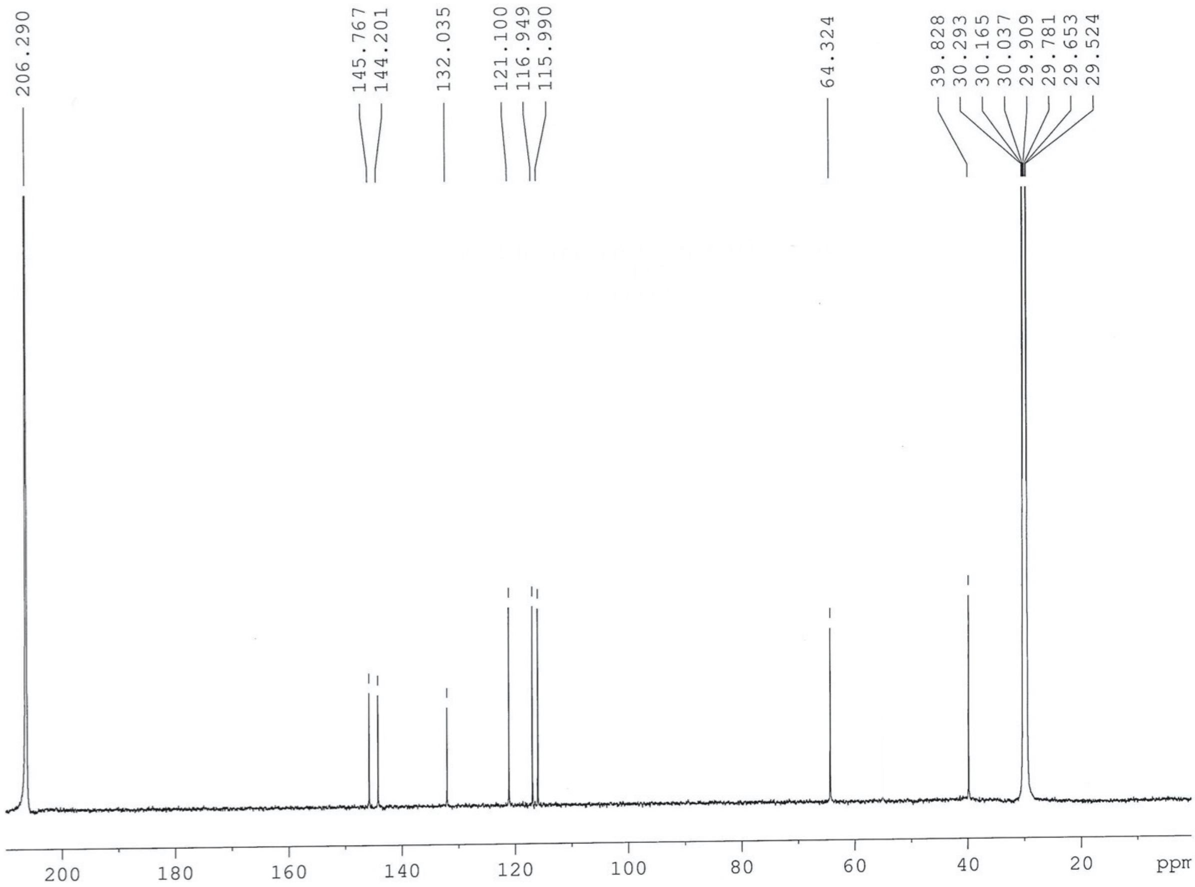

Figure S10. ^{13}C NMR in $(CD_3)_2CO$ hydroxytyrosol

Bibliography

1. Pérez-Trujillo, M.; Gómez-Caravaca, A.M.; Segura-Carretero, A.; Fernández-Gutiérrez, A.; Parella, T. Separation and Identification of Phenolic Compounds of Extra Virgin Olive Oil from Olea europaea L. by HPLC-DAD-SPE-NMR/MS. Identification of a New Diastereoisomer of the Aldehydic Form of Oleuropein Aglycone. *J. Agric. Food Chem.* **2010**, *58*, 9129–9136, doi:10.1021/jf101847e.
2. Bianco, A.; Uccella, N. Biophenolic components of olives. *Food Res. Int.* **2000**, *33*, 475–485, doi:10.1016/S0963-9969(00)00072-7.
